# Supplementary material for: Membrane curvature induced by proximity of anionic phospholipids can initiate endocytosis
Source: Nat Commun. 2017 Nov 9;8:1393. doi: 10.1038/s41467-017-01554-9 (PMC5680216; doi:10.1038/s41467-017-01554-9)
Supplement: Supplementary file 1 — Supplementary Information [file 41467_2017_1554_MOESM1_ESM.pdf]

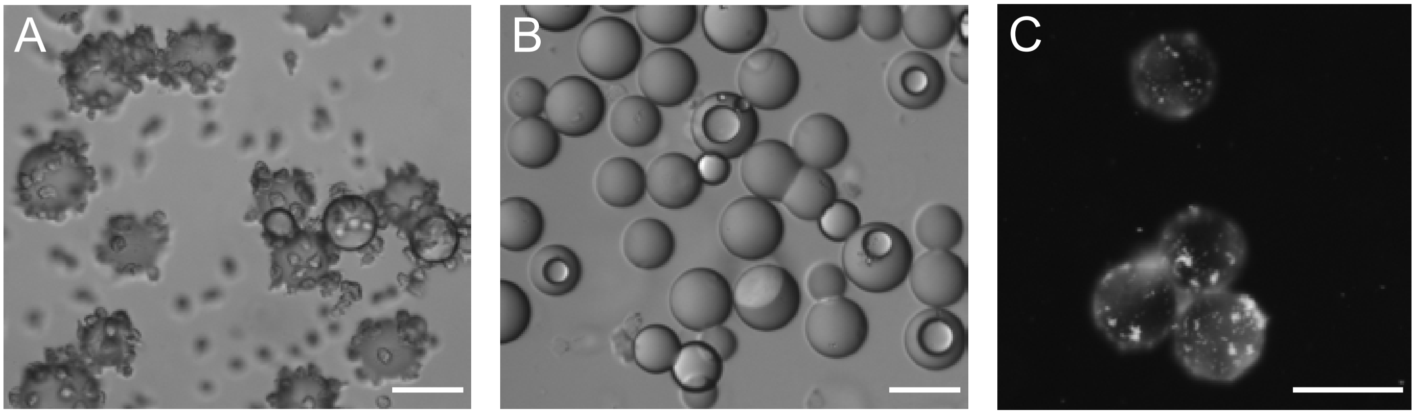

Supplementary Figure 1. Isolation of plasma membranes from HeLa cells
(A) Suspended HeLa cells were initially allowed to attach to polycationic beads (Affi-gel 731, Bio-Rad). (B) To shear the cells off while retaining the adherent plasma membranes on the beads, the suspension was subjected to vigorous vortexing in the presence of heparin, to block binding of internal membranes and other cellular contents to the beads. (C) Visualization of the adherent isolated membranes by staining with the fluorescent lipid dye DiI.

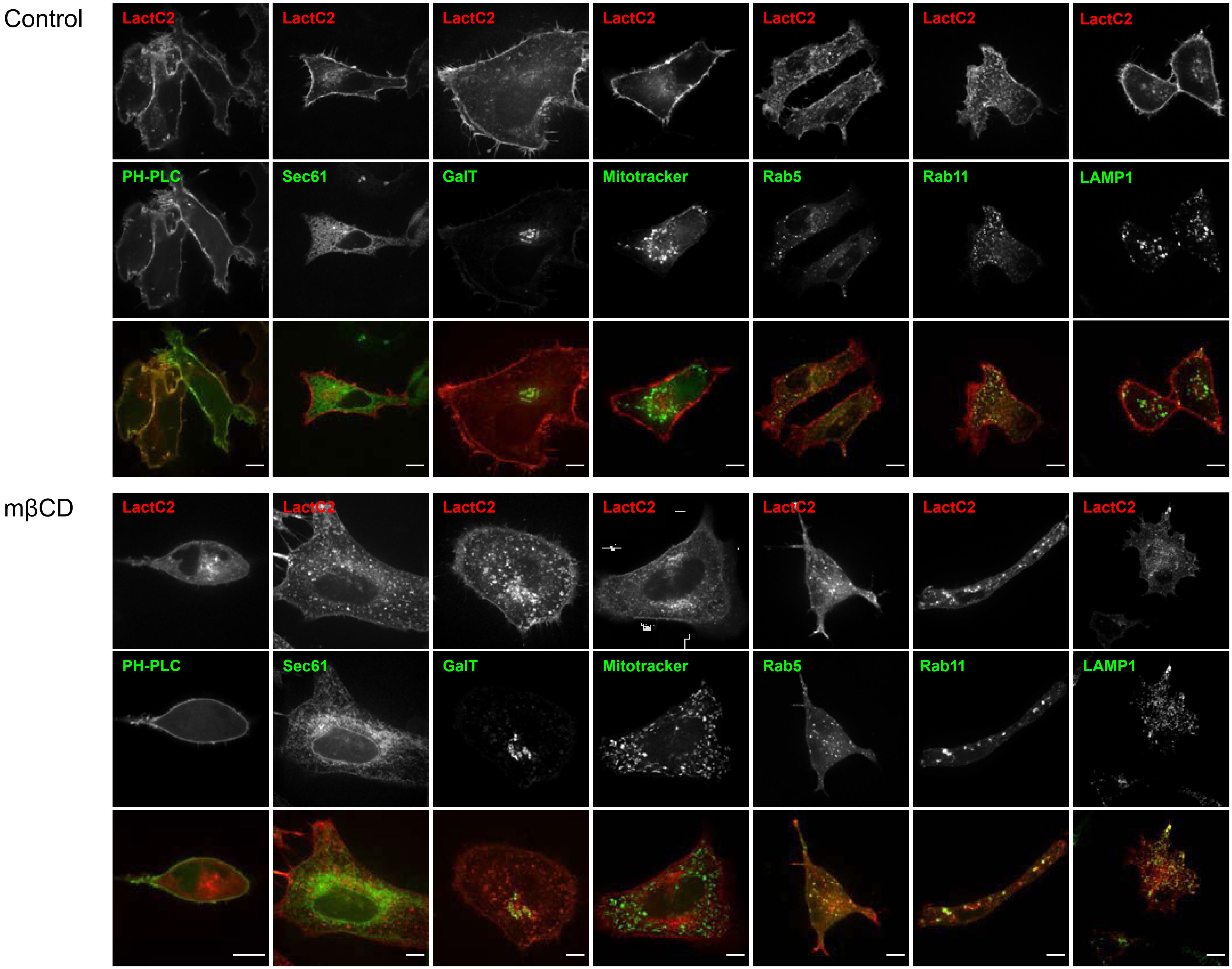
Control
LactC2
LactC2
LactC2
LactC2
LactC2
LactC2
LactC2
PH-PLC
Sec61
GalT
Mitotracker
Rab5
Rab11
LAMP1
mβCD
LactC2
LactC2
LactC2
LactC2
LactC2
LactC2
LactC2
PH-PLC
Sec61
GalT
Mitotracker
Rab5
Rab11
LAMP1

Supplementary Figure 2.Subcellular distribution of PtdSer with respect to various organellar markers

Control HeLa cells are shown in top section, while mβCD-extracted cells are shown at bottom. Images are representative of the experiments used to calculate the Pearson's coefficients documented in Figure 1G. Scale bar, 10 μm

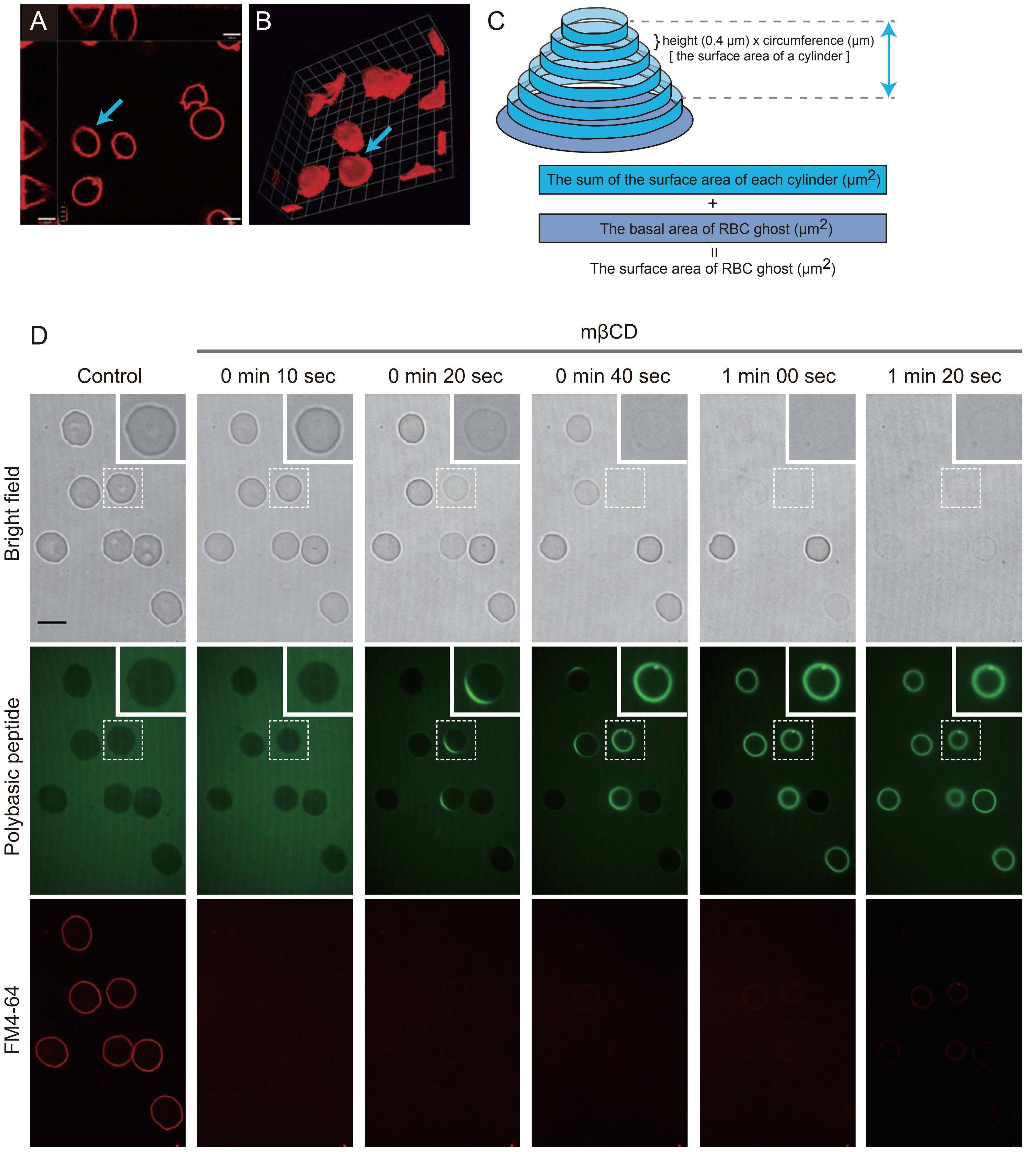

A
B
C
} height (0.4 μm) x circumference (μm)
[the surface area of a cylinder]
The sum of the surface area of each cylinder (μm2)
+
The basal area of RBC ghost (μm2)
=
The surface area of RBC ghost (μm2)
D
mβCD
Control
0 min 10 sec
0 min 20 sec
0 min 40 sec
1 min 00 sec
1 min 20 sec
Bright field
Polybasic peptide
FM4-64

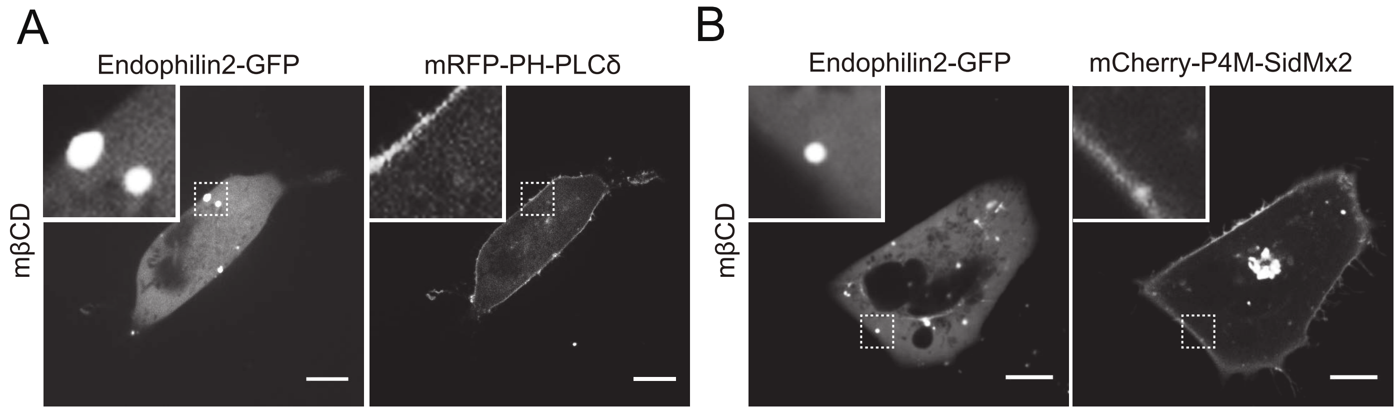

Supplementary Figure 4. Endophilin positive foci and depleted in PtdIns4P and PtdIns4,5P$_2$
Confocal images of HeLa cells following a 2 min treatment with mβCD co-transfected with Endophilin2-GFP and either the PtdIns4,5P2 probe GFP-PLCδ-PH (A) or the PtdIns4P probe GFP-P4M-SidMx2 (B). Insets: magnification of the areas indicated by a square.

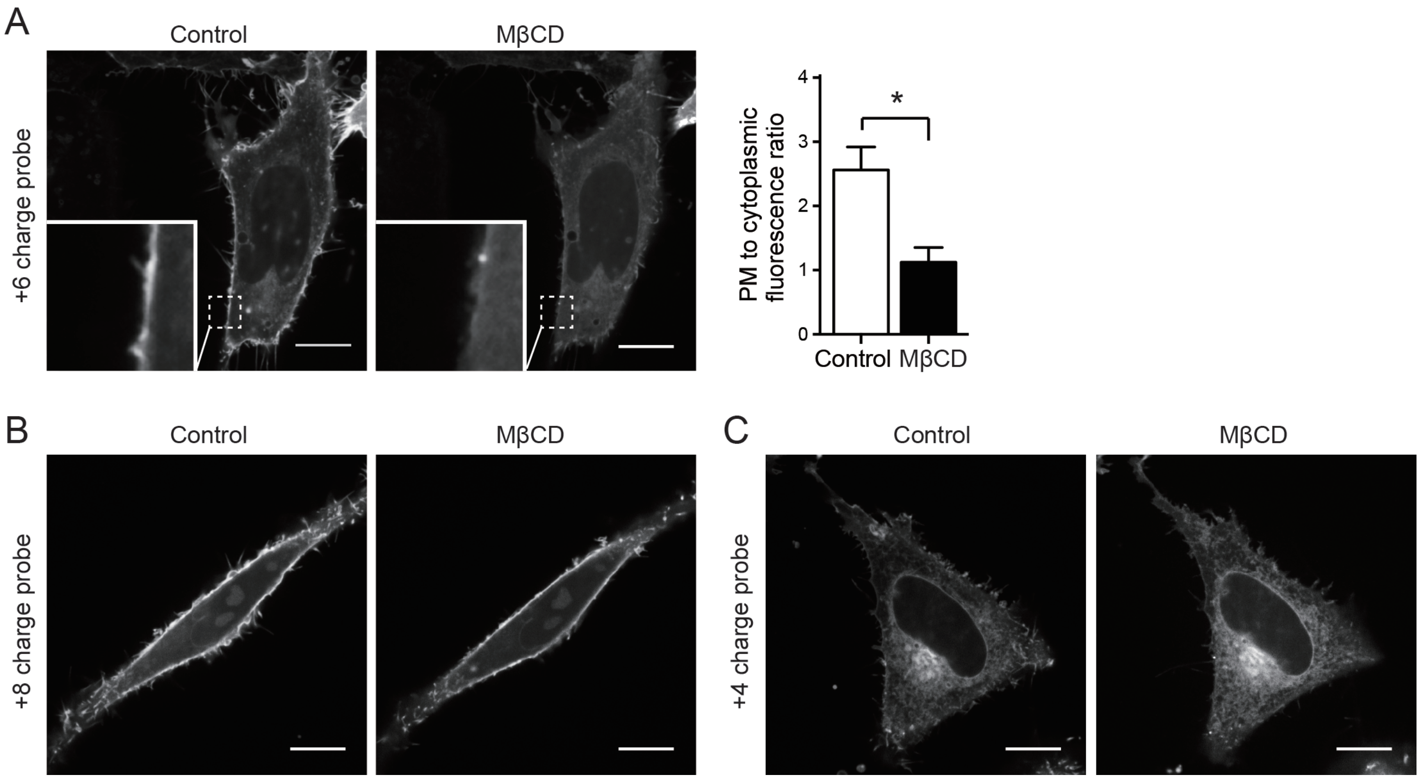

Supplemental Figure 5. Redistribution of membrane charge probes following Cholesterol extraction. Hela cells transfected with the indicated charge probes before and 10 min after treatment with mbCD. The +6 probe (panel A) is partly depleted from the plasma membrane. (B, C) The impact on the +4 and +8 probes is more subtle. Values represent means +/- s.e.m., n=12.

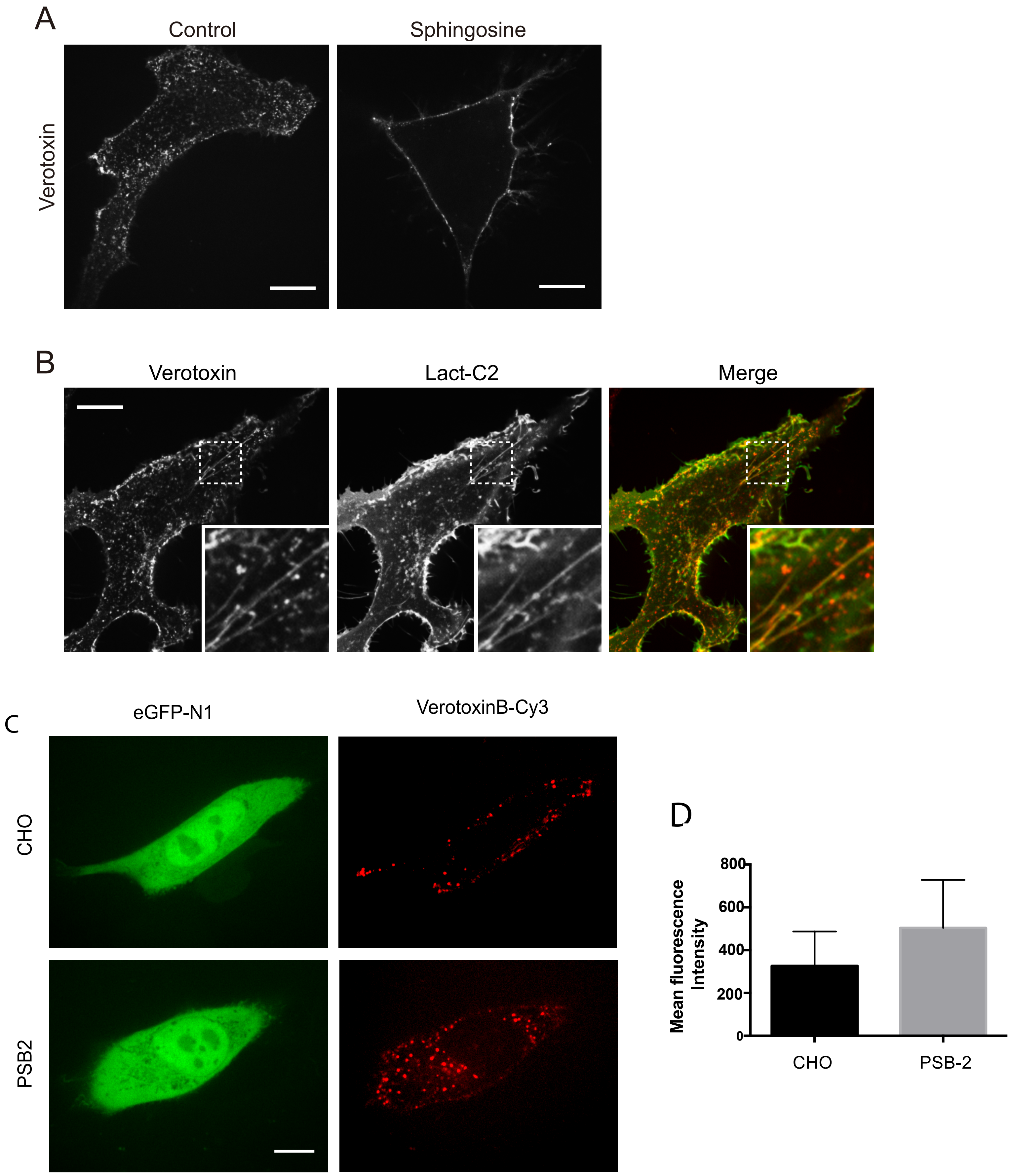
A
Control
Sphingosine
Verotoxin
B
Verotoxin
Lact-C2
Merge
C
eGFP-N1
VerotoxinB-Cy3
CHO
PSB2
D
Mean fluorescence Intensity
800
600
400
200
0
CHO
PSB-2

Supplementary Figure 6. Verotoxin-induced endocytosis is inhibited by charge neutralization but does not specifically require phosphatidylserine.

(A) Recombinant vertoxin (Shiga-like toxin) B-subunit induces endocytosis in HeLa cells. HeLa cells incubated with verotoxin-Cy3 (0.25 μg/ml) for 15 min at 37°C induces internalization via a tubular endocytic carrier. Pre-incubation with 10 μM sphingosine, a positively charged lipid, for 5 min prior to the addition of vertoxin prevents the internalization of verotoxin but not binding to its ligand globotriaosylceramide, Gb3. (B) Tubulo-vesicular carriers induced by verotoxin are positive for PtdSer as monitored by the Lact-C2 probe. (C-D) PSB-2 cells have a ~95% reduction in PtdSer synthase activity due to a ~75% reduction in PtdSer. PSB-2 and the parental CHO cells do not normally synthesize Gb3. However, transient expression of the Gb3 synthase, α1,4-Glycosyltransferase (A4GALT), is sufficient to produce Gb3. CHO or PSB-2 cells were co-tranfected with pCMV-SPORT-A4GALT and pEGFP (3:1) to ensure that GFP positive cells also express the Gb3 synthase. Transfected cells were incubaed with verotoxin-Cy3 as in panel (A). Values represent means +/- s.e.m., n=40. Verotoxin is internalized by both cells types. *Please note:* with a 75% reduction in PtdSer, the PSB-2 cells produce more phosphatidylinositol another anionic lipid and presursor of the more anionic phosphorylated phosphoinositides to compensate for the loss of negative charge.

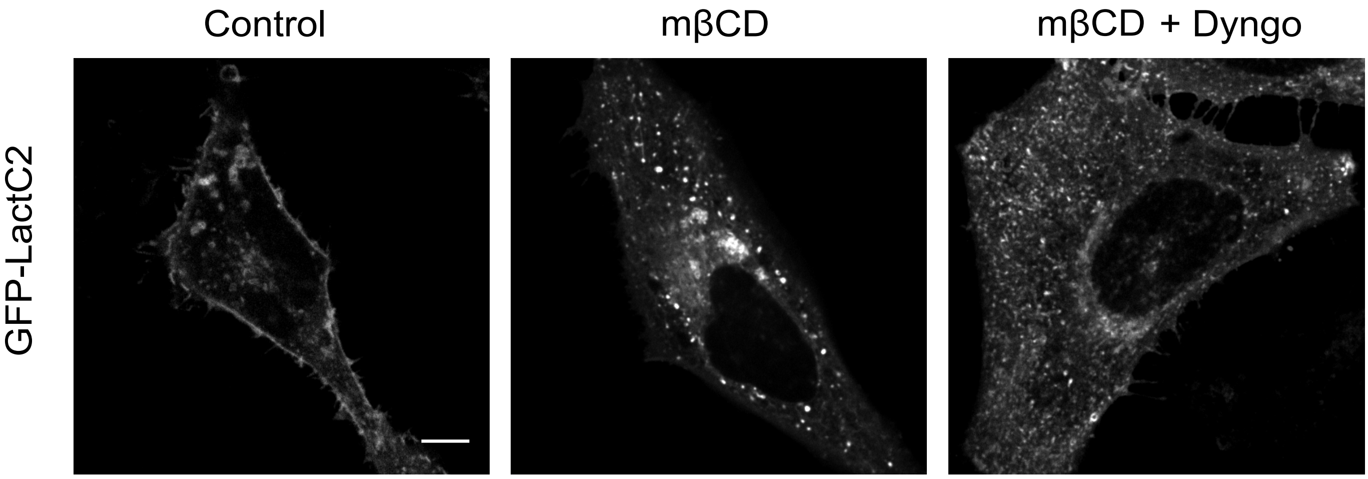

Supplementary Figure 7.Dynamin is not required for mβCD-stimulated endocytosis. HeLa cells expressing GFP-Lact-C2 were treated with 10 mM mβCD in the presence or absence of 30 μM of Dyngo4a. Images are representative of three separate experiments. Scale bar, 10 μm

Supplementary Methods

Description of the system

We consider a lipid bilayer consisting of two monolayers.
Each monolayer has an overall surface charge, which comes from all charged lipids: PtdSer, PtdIns(4,5)P_2 etc. The charge densities (charge per unit area) in the outer and inner monolayers will be denoted by σ_{out} and σ_{in}, respectively.

Each monolayer resists bending deformation quantified by the total curvature, J, (the sum of two principle curvatures) of the monolayer surface.

The bending energy per unit area of the monolayer surface, f_B, is determined by Helfrich model as

$$f_B = \frac{1}{2}\,\kappa\, J^2 \qquad (1)$$

The aqueous solution bathing the bilayer is be characterized by the dielectric constant, ε, and t the concentration, c_0, of 1:1 electrolyte (ionic strength). The characteristic length of charge screening within the solution referred to as Debay length, λ_D, is given by

$$\lambda_D = \sqrt{\frac{\varepsilon\,\varepsilon_0\,k_B T}{2e^2\,c_0}} \qquad (2)$$

where ε_0 is the dielectric constant of vacuum, $k_B T$ is the product of Boltzmann constant and the absolute temperature, and e is the elementary charge. For the physiological ionic strength $c_0 = 100$mM, the Debay length is λ_D=1nm.

Spontaneous curvature of monolayer

The electrostatic free energy of each monolayer, related to its charge density, σ, results in a monolayer tendency to bend such that the monolayer surface adopts a convex shape (Scheme 1).

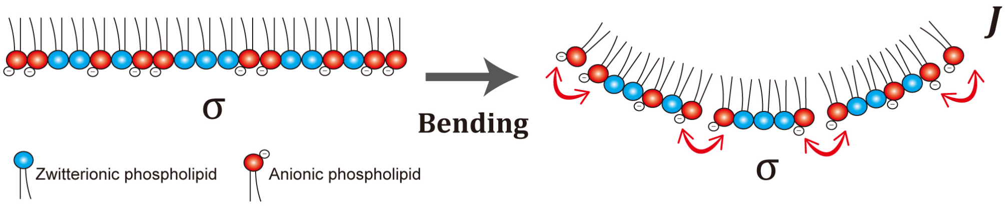

Scheme 1 Spontaneous monolayer curvature

The curvature of this favorable monolayer shape is referred to as the spontaneous curvature, J_S. We define the curvature as illustrated in Scheme 1 to be positive. The previous work suggests that, for moderate surface charge densities, the electrostatically induced spontaneous curvature of a monolayer is given by

$$J_S = \frac{1}{4}\frac{\sigma^2\,\lambda_D^2}{\varepsilon\,\varepsilon_0\,\kappa}\left(1+\frac{2\delta}{\lambda_D}\right) \qquad (3)$$

where $\delta \sim 0.5nm$ is the distance between the level of the glycerol backbone within the monolayer representing the monolayer neutral surface and the effective plane of the headgroup electric charges.

For the following, it is convenient to express the monolayer spontaneous curvature through the fraction, ϕ, of charged lipids within the monolayer,

$$J_S = \frac{1}{4}\frac{\lambda_D^2\,e^2}{\varepsilon\,\varepsilon_0\,\kappa\,a^2}\left(1+\frac{2\delta}{\lambda_D}\right)\phi^2\,. \qquad (4)$$

where a is the area per lipid in the monolayer plane and it is assumed that a charged lipid polar head carries lipids carries one elementary charge, e .

Using the values $\delta = 0.5nm$, $\lambda_D = 1\text{nm}$, , $\varepsilon = 80$, $\varepsilon_0 = 8.9\ 10^{-12}$ F/m, $e \sim 1.6\ 10^{-19}\ C$, $\kappa \sim 10\ k_B T$, $k_B T \sim 4\ 10^{-21}$Joule, and $a = 0.7\ nm^2$, we obtain for the monolayer spontaneous curvature,

$$J_S = 0.92\,\phi^2\,\frac{1}{\text{nm}} \qquad (5)$$

Alternatively, the radius of a cylinder such a monolayer tends to form is

$$R_S = 1.1\,\frac{1}{\phi^2}\,\text{nm} \qquad (6)$$

Spontaneous curvature of a bilayer

The spontaneous curvature of a bilayer, J_B, is determined by the spontaneous curvatures of its inner, J_S^{in}, and outer, J_S^{out} , monolayer. In the approximation of moderate curvatures it can be presented as

$$J_B = \frac{1}{2}\left(J_S^{in} - J_S^{out}\right) \qquad (7)$$

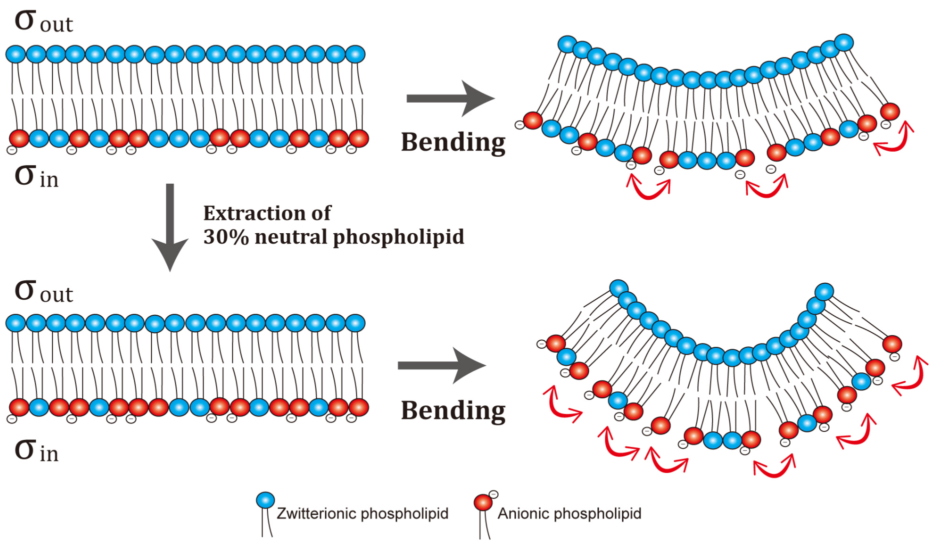

Scheme 2 Spontaneous bilayer curvature

Using Eqs.(5),(7), and we obtain for the bilayer spontaneous curvature

$$J_B = 0.46\,(\phi_{in}^2 - \phi_{out}^2)\,\frac{1}{\text{nm}} \quad (8)$$

or the radius of a preferable bilayer cylinder

$$R_B = 2.2\,\frac{1}{\phi_{in}^2 - \phi_{out}^2}\ \text{nm} \quad (9)$$

Conclusion

Eqs.8 and 9 represent the major result of the computation, which can be used to estimate the membrane spontaneous curvature depending on the fraction of charged lipids in the inner and outer monolayer. Once we have numbers for ϕ_{in} and ϕ_{out} before and after the cholesterol extraction, we can estimate the corresponding spontaneous curvatures.

For the moment, let us make a simplified estimation for the fraction of charged lipids in the inner monolayer, ϕ_{in}, needed to generate tubules of 25nm radius (50nm diameter). Assume, for simplicity, that the outer monolayer is absolutely neutral, $\phi_{out} = 0$. We get that the required ϕ_{in}=0.3.
