# Supplementary material for: Membrane curvature induced by proximity of anionic phospholipids can initiate endocytosis
Source: Nat Commun. 2017 Nov 9;8:1393. doi: 10.1038/s41467-017-01554-9 (PMC5680216; doi:10.1038/s41467-017-01554-9)
Supplement: Supplementary file 3 — Description of Additional Supplementary Files [file 41467_2017_1554_MOESM3_ESM.pdf]

Description of Additional Supplementary Files

File Name: Supplementary Movie 1

Description: **Structured Illumination Microscopy of Endophilin2- GFP in control HeLa cells.** HeLa cells expressing endophilin2-GFP were fixed with 4% PFA for 15 minutes. Images were acquired using SIM as described in the Methods and reconstructed using the structured illumination module in the ZEN software. 3-D SIM image and animation were constructed using the Imaris software.

File Name: Supplementary Movie 2

Description: **Structured Illumination Microscopy of Endophilin2- GFP in cholesterol extracted HeLa cells.** HeLa cells expressing endophilin2-GFP were treated with 10 mM mβCD for 10 min at 37 °C and subsequently fixed with 4% PFA for 15 minutes. Images were acquired using SIM as described in the Methods and reconstructed using the structured illumination module in the ZEN software. 3-D SIM image and animation were constructed using the Imaris software.
